# HIV-1 self-testing to improve the efficiency of pre-exposure prophylaxis delivery: a randomized trial in Kenya

**DOI:** 10.1186/s13063-019-3521-2

**Published:** 2019-07-04

**Authors:** Katrina F. Ortblad, John E. Kearney, Kenneth Mugwanya, Elizabeth M. Irungu, Jessica E. Haberer, Ruanne V. Barnabas, Deborah Donnell, Nelly Rwamba Mugo, Jared M. Baeten, Kenneth Ngure

**Affiliations:** 1 Department of Global Health, University of Washington, 908 Jefferson St, Seattle, WA 98104 USA; 2 Massachusetts General Hospital, Boston, USA; 3 Harvard Medical School, Boston, USA; 4 Department of Medicine, University of Washington, Seattle, USA; 5 Fred Hutchinson Cancer Research Center, Seattle, USA; 6 Center for Clinical Research, Kenya Medical Research Institute, Nairobi, Kenya; 7 Department of Epidemiology, University of Washington, Seattle, USA; 8 Department of Community Health, Jomo Kenyatta University of Agriculture and Technology, Nairobi, Kenya

**Keywords:** HIV-1 testing, HIV-1 self-testing, Kenya, Randomized trial, PrEP, HIV-1 serodiscordant couples, Young women, Delivery models

## Abstract

**Background:**

The introduction of pre-exposure prophylaxis (PrEP) for human immunodeficiency virus-1 (HIV-1) prevention in Africa presents new challenges for health systems that are already overburdened because PrEP delivery requires frequent clinic visits (generally every 3 months) for HIV-1 testing and PrEP refills. HIV-1 self-testing (HIVST) has the potential to improve the efficiency of PrEP delivery by decreasing the number of clinic visits. Here, we describe the rationale and design of a randomized, noninferiority trial designed to test the effectiveness and safety of using HIVST to support PrEP delivery in Kenya.

**Methods:**

The JiPime-JiPrEP (Kiswahili for ‘Test Yourself, PrEP Yourself’) study is a three-arm randomized trial taking place in Thika, Kenya. Participants (*n* = 495) are eligible for enrollment if they are at least 18 years old, HIV-1 seronegative, and have been taking PrEP for 1 month. Three distinct participant types will be enrolled: men (*n* = 165) and women (*n* = 165) who are in mutually disclosed HIV-1 serodiscordant relationships, and women (*n* = 165) who are at HIV-1 risk and not in a known serodiscordant relationship. Participants in each of these subpopulations will be 1:1:1 randomized to: 1) the standard of care, with quarterly clinic visits; 2) oral HIVST, with biannual clinic visits plus oral HIVSTs to use at the quarters between those visits; or 3) blood-based HIVST, with biannual clinic visits plus blood-based HIVSTs. All participants will complete quantitative surveys and provide blood samples for the objective measurement of PrEP adherence at baseline, 6 months, and 12 months. The primary outcomes are PrEP adherence, PrEP continuation, and HIV-1 testing, measured at 6 months and secondarily at 12 months.

**Discussion:**

The findings from this trial can help to understand how HIVST—a new HIV-1 testing technology—can support health systems in sub-Saharan Africa. Additionally, the findings can inform policy aimed at improving the efficiency of PrEP implementation and scale-up in Kenya.

**Trial registration:**

ClinicalTrials.gov, NCT03593629. Retrospectively registered on 20 July 2018.

**Electronic supplementary material:**

The online version of this article (10.1186/s13063-019-3521-2) contains supplementary material, which is available to authorized users.

## Background

More than two million individuals become newly infected with human immunodeficiency virus-1 (HIV-1) each year, with the majority being in sub-Saharan Africa [1]. In Kenya, more than 1.4 million people are living with HIV-1 [2], making it the country with the fourth highest number of persons living with HIV-1. The past 5 years have witnessed major strides in the development of highly effective HIV-1 prevention interventions, particularly those using antiretroviral medications, namely, antiretroviral therapy (ART) for HIV-1 infected persons to decrease infectiousness and pre-exposure prophylaxis (PrEP) for uninfected persons to prevent acquisition. Novel strategies for delivering these highly effective interventions are needed to achieve maximum impact among populations at the highest risk of HIV-1.

PrEP has been shown to be efficacious and safe for reducing HIV-1 risk among men who have sex with men [3, 4], heterosexual men and women [5–7], pregnant women [8], and injection drug users [9] in diverse geographic settings. As with ART, adherence is essential for PrEP efficacy. PrEP clinical trials found a wide range of PrEP efficacy results [10], as explained by the degree to which the trial populations were adherent to PrEP [11]. Secondary analyses from these trials and PrEP demonstration studies have shown that, at the individual level, HIV-1 protection is about 90–100% when PrEP adherence is high [5, 12–14]. In the PrEP demonstration studies, HIV-1 incidence among PrEP users was low and PrEP clinic visits were brief [15], suggesting that PrEP users may not need frequent or intensive follow-up to achieve high PrEP adherence [15].

PrEP delivery can be expensive in terms of medication costs, staffing time, laboratory testing, and patient opportunity costs [16, 17]. In costing analyses in East Africa, it was estimated that adding PrEP to routine public health services using Ministry of Health personnel, drugs, and laboratories would add approximately USD $100 annually per person. Notably, in those models, the greatest proportion of the total costs was not the cost of medication but was instead the cost of personnel (39% of the cost) [18]. PrEP can also be costly for clients, as time away from work and travel costs associated with PrEP clinic visits can be an economic burden [19, 20].

PrEP requires regular HIV-1 testing, both at the time of PrEP initiation and then quarterly thereafter [15, 21, 22]. HIV-1 testing is necessary to reduce the risk of ART resistance if HIV-1 infection is present prior to PrEP or in the rare instances of HIV-1 breakthrough infections [23–25]. As PrEP expands to public health clinics in Africa, new and improved models of HIV-1 testing while on PrEP are needed. HIV-1 self-testing (HIVST) has been shown to be acceptable among diverse populations [26–31]. Self-testing offers strong advantages over clinic-based HIV-1 testing, including increased privacy and convenience for patients, and reduced clinic flow for providers. To our knowledge, no HIV-1 clinics distributing PrEP have attempted to move HIV-1 testing out of the clinic and into the home with HIVST; evidence is needed to motivate policy change for this opportunity.

The present study, titled JiPime-JiPrEP (Kiswahili for ‘Test Yourself, PrEP Yourself’*)*, proposes that the delivery of HIVST with PrEP can reduce the frequency of clinic visits and clinic-based HIV-1 testing among persons receiving PrEP, and can do so safely and without diminishing PrEP adherence. The proposed model has several potential advantages. First, the model could help achieve the objectives of achieving high PrEP adherence and high HIV-1 testing coverage, without increasing the clinic costs and client burden of PrEP delivery. Second, both PrEP and HIVST are new in Kenya (both were introduced formally in May 2017) [32] and seeing how they fit together in a novel HIV-1 prevention intervention is an exciting opportunity. Third, increased efficiency of PrEP delivery could be appealing to not only policy makers and health providers [33], but also to PrEP users.

## Methods and analysis

### Study design

**Fig. 1:**
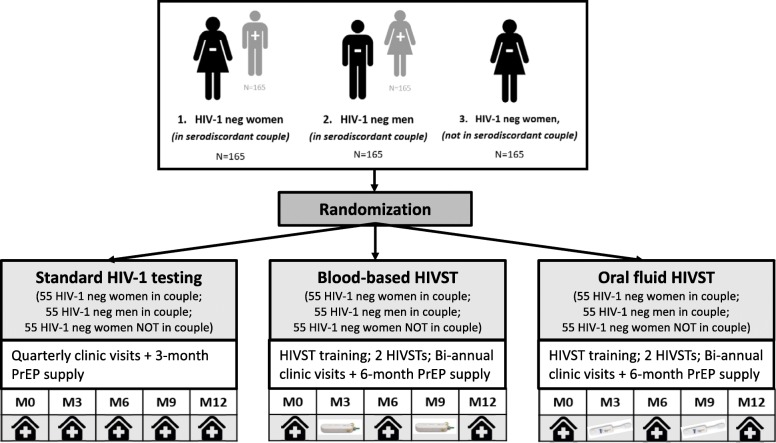
Flow diagram of study enrollment, randomization, and intervention arms. Note that all participants will receive HIV-1 counseling, condoms, and sexually transmitted infection management at clinic visits. *HIVST* HIV-1 self-testing, *neg* negative, *PrEP* pre-exposure prophylaxis.

JiPime-JiPrEP is a three-arm randomized, noninferiority trial (ClinicalTrials.gov identifier: NCT03593629) [34], designed to test the use of HIVST to decrease the frequency of clinic visits for PrEP while resulting in equivalent PrEP adherence and HIV-1 testing (Fig. 1; the Standard Protocol Items: Recommendations for Interventional Trials (SPIRIT) checklist is provided as Additional file 1 [35]). The trial will test the effect of a novel PrEP delivery model on three distinct populations at risk of HIV-1: 1) HIV-1 uninfected men who are in a relationship with an HIV-1 infected woman (i.e., HIV-1 serodiscordant relationship) (*n* = 165); 2) HIV-1 uninfected women in an HIV-1 serodiscordant relationship (*n* = 165); and 3) HIV-1 uninfected women not known to be in an HIV-1 serodiscordant relationship (*n* = 165) who are at increased risk of HIV-1 infection compared to HIV-1 uninfected men not in an HIV-1 serodiscordant relationship. Within each population subtype, participants will be randomized in a 1:1:1 fashion to one of the following study arms: 1) standard-of-care, with quarterly clinic visits and a 3-month PrEP supply; 2) oral HIVST, with biannual clinic visits and a 6-month PrEP supply plus two oral HIVSTs to be used at quarters between clinic visits; and 3) blood-based HIVST, with biannual clinic visits and a 6-month PrEP supply plus two blood-based self-tests for use between clinic visits. This study includes different study arms for oral and blood-based HIVST to help determine participants’ HIVST preferences. Participants will be followed for 1 year, with follow-up assessments at 6 and 12 months (participants in the standard-of-care group will additionally be followed up at 3 and 9 months [36]).

### Specific aims

The specific aims of this trial are to test the use of HIVST and PrEP delivery on: 1) PrEP adherence (measured objectively by detection of PrEP medication in blood samples); 2) the persistence in PrEP refilling; and 3) HIV-1 testing among PrEP users. Additionally, the trial design will enable us to: 1) measure the impact of two different HIVST modalities, oral-fluid and blood-based, to support PrEP delivery; and 2) evaluate the effect of HIVST and PrEP delivery among various subpopulations (women and men in HIV-1 serodiscordant relationships, and women at HIV-1 risk, including those in and not in serodiscordant relationships).

### Study oversight

A Data Safety Monitoring Board (DSMB) will oversee the data collected as part of this study every 6 months. The DSMB will contain members from the United States and Kenya with expertise in HIV-1 epidemiology, statistics, ethics, and PrEP delivery in high HIV burden settings. The DSMB will review study execution, PrEP adherence, HIV-1 incidence, HIV-1 drug resistance, and reported serious adverse events and social harms in both open and closed sessions, and provide recommendations to the study team.

### Setting

The study will take place in at the Partners in Health and Research Development (PHRD) clinic located in Thika, Kenya. Thika is an urban center about 40 km outside of Nairobi, with a large surrounding peri-urban and rural population. The PHRD clinic has established a multidisciplinary team to conduct HIV-1 and sexually transmitted disease prevention research and provide clinical care, particularly around HIV-1 testing, HIV-1 comprehensive care, and HIV-1 prevention services. This team includes trained and certified counselors, physicians, pharmacists, and laboratory technicians, as well as experienced quantitative and qualitative researchers, implementation scientists, and data management specialists. The PHRD clinic has been involved in PrEP clinical and implementation research since its founding in 2006 and members of this team were deeply involved in the development of Kenya’s guidelines for PrEP delivery [14, 20, 36–42]. The PHRD clinic is a center of excellence for PrEP in Kenya and is leading the training of other clinics as part of Kenya’s national PrEP scale-up [43].

### Recruitment

Participants will be recruited from Kiambu County, which contains the PHRD clinic, as well as from neighboring counties. The PHRD clinic has established successful recruitment strategies, which include collaborating with existing HIV-1 testing centers, and community-based mobilization to engage couples and women in HIV-1 prevention. Participants will be assessed for eligibility only after being enrolled on PrEP for 1 month at a Kenyan Ministry of Health (MOH) approved clinic. A number of people start PrEP out of curiosity; thus, this approach will allow participants likely to persist sufficiently to assess study aims. Formal eligibility determination will be done in the PHRD clinic by a trained counselor.

#### Inclusion and exclusion criteria

Complete inclusion and exclusion criteria are summarized in Table 1. All participants must be 18 years of age or older, willing to complete a rapid HIV-1 test, and willing to provide written informed consent.

**Table 1:** Study inclusion and exclusion criteria

Inclusion criteria	Exclusion criteria
▪ 18 years of age or older	▪ Less than 18 years old
▪ Test HIV-1 negative (blood-based rapid HIV-1 test)	▪ Test HIV-1 positive (blood-based rapid HIV-1 test)
▪ Has been taking PrEP for at least 1 month prior to enrollment and planning to continue on PrEP	▪ Not currently taking PrEP
▪ Willing to be randomized to either clinic-based HIV-1 testing or at-home HIVST	▪ Unwilling to be randomized to either clinic-based testing or at-home HIVST
▪ Not currently enrolled in an HIV-1 prevention clinical trial	▪ Enrolled in an HIV-1 prevention clinical trial
	▪ Contraindication to use of tenofovir disoproxil fumarate/emtricitabine (TDF/FTC) or TDF/lamivudine (TDF/3TC)
▪ Able and willing to provide written informed consent	▪ Unable or unwilling to provide written informed consent

Note that women who are pregnant or become pregnant during the study are still eligible for study participation

*HIV-1* human immunodeficiency virus-1, *HIVST* HIV-1 self-testing, *PrEP* pre-exposure prophylaxis

### Study procedures

**Fig. 2:**
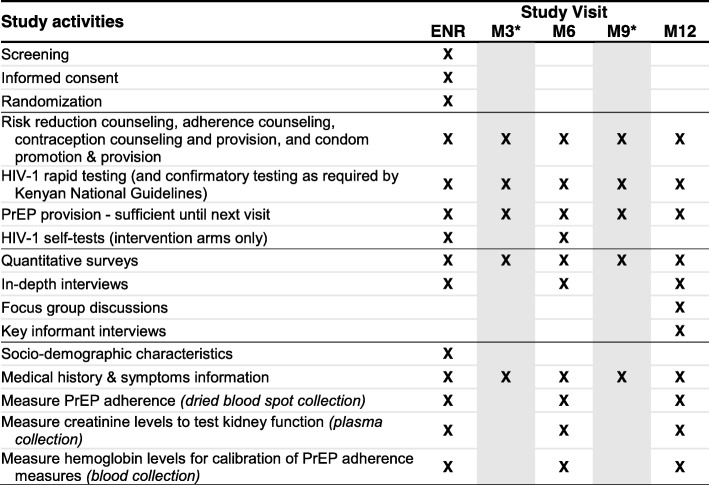
Schedule of enrollment, interventions, and assessments for the JiPime-JiPrEP randomized trial. *Only participants randomized to the standard-of-care arm have visits at months 3 and 9. *ENR* enrollment, *M* month, *PrEP* pre-exposure prophylaxis

An overview of study procedures and activities is summarized in Fig. 2.

#### Study visits

At each study visit, participants will meet with study counselors in private clinic counseling rooms. At screening, counselors will assess potential participants for eligibility (Table 1)**,** and those who meet the eligibility requirements will be guided through the informed consent procedures and enrolled in the study. At each study visit, participants will receive counseling as per national guidelines on HIV-1 risk reduction practices, PrEP adherence, and family planning methods. All participants will be offered rapid blood-based HIV-1 testing (with associated pre- and post-test counseling) at each study visit and interested participants will be offered urine pregnancy testing and condoms. As per the Kenyan national PrEP guidelines, the renal function of participants on PrEP will be measured annually [36]. In addition, participant’s blood hemoglobin levels will be tested at 6 and 12 months to standardize measures of PrEP in dried blood spot (DBS) samples. At each study visit, the clinicians will perform a physical assessment and collect study samples. Finally, pharmacists will dispense PrEP bottles (the number of which will depend on the participant’s intervention arm) and HIVSTs (to participants in the intervention arms). Study counselors will encourage participants to use PrEP as long as they are at risk of HIV-1 infection and recommend that participants in HIV-1 serodiscordant relationships discontinue PrEP after their partner living with HIV has used ART for ≥ 6 months (aligning with expected viral suppression) and there are no concerns about ART adherence or additional sex partners.

#### Randomization

Participants within each subpopulation will be individually randomized to one of three study arms. The study randomization list will be created using variable-sized blocks. Study arm assignments will be placed in opaque envelopes labeled with preassigned participant IDs, specific to each subpopulation type. Study pharmacists will conduct individual-level randomization at the first study visit after participants complete the baseline survey and physical assessment. The participant being randomized will open the randomization envelope that matches the participant’s ID number assigned to them with a study pharmacist, so that the participant can be assured of a fair randomization process. The study will be unblinded after random assignment.

#### Standard-of-care arm

Participants assigned to the clinic-based standard-of-care arm will be administered a 3-month PrEP drug supply—three bottles of tenofovir disoproxil fumarate/emtricitabine (TDF/FTC) or TDF/lamivudine (TDF/3TC) to be taken daily—by a study pharmacist and they will be instructed to return to the PHRD clinic for PrEP refills and clinic-based HIV-1 testing once they complete all their PrEP bottles. The PrEP drugs issued in this study will reflect those that are being offered free by the Kenyan MOH as part of the national PrEP scale-up.

#### HIVST intervention arms

Participants assigned to one of the two HIVST intervention arms will be trained by a study pharmacist on how to use the HIVST they are randomized to receive. Training will include watching an HIVST tutorial video [44, 45] produced by the HIVST manufacturers on a tablet (language options include either English or Kiswahili), discussing the HIVST manufacturers’ instructions with the pharmacists, having the opportunity to use an HIVST, and asking questions in the presence of the pharmacist. Once trained on HIVST, pharmacists will give participants two self-tests (that match the HIVST arm to which they are randomized) and a 6-month PrEP drug supply. The pharmacists will instruct participants to test for HIV-1 using HIVST once they complete their third PrEP bottle, and to return to the research clinic for PrEP refills and clinic-based HIV-1 testing once they complete all bottles.

This study will use two different HIVSTs: the OraQuick In-Home HIV-1 Test (OraSure Technologies, Bethlehem, USA) for oral-fluid HIVST, and the AtomoRapid™ HIV (1&2) Test (Atomo Diagnostics, Oxfordshire, UK) for blood-based HIVST. The OraQuick In-Home HIV Test tests for HIV-1 and HIV-2 antibodies in oral fluid, gives results in about 20 min, and has 91.7% sensitivity and 99.9% specificity [46]. The AtomoRapid™ HIV (1&2) Test tests for HIV-1 and HIV-2 antibodies in the blood via a finger prick, gives results in 15 min, and has 99.8% sensitivity and 100% specificity [45].

### Data collection

Over the 12-month duration of the study, quantitative data (i.e., surveys) will be collected from all participants and qualitative data (i.e., in-depth individual interviews and focus group discussions (FGDs)) will be collected from a subset of participants and key informants (e.g., PrEP providers) (Fig. 2).

#### Quantitative data

All participants will complete surveys at enrollment, 6 months, and 12 months. At enrollment, counselors will collect basic demographic information. At all study visits, counselors, clinicians, and pharmacists will collect information on sexual behaviors, fertility desires, alcohol and substance use, depression indicators, general self-efficacy, medical history, PrEP adherence, HIVST preferences, and HIV-1 serodiscordant relationship dynamics (if applicable). At the end of the study (month 12), participants’ HIVST preferences will be assessed after they have the opportunity to use an HIVST method they did not previously have access to at the clinic (i.e., participants in the standard-of-care arm will get the opportunity to use both oral-fluid and blood-based HIVSTs, and those randomized to HIVST will have the chance to use the other HIVST modality). Extra efforts (e.g., home visits) will be made to follow-up with participants who have not arrived 3 months after their scheduled 12-month visit date so that DBS samples can be collected to measure PrEP adherence. All survey data will be collected in private rooms using face-to-face interviews and CommCare (Dimagi, Cambridge, USA); an electronic data collection platform that allows real-time data monitoring.

#### Qualitative data

A subset of participants (*n* = 20 from each subpopulation, 60 in total, selected across randomization arms) will complete serial in-depth interviews at enrollment, 6 months, and 12 months, and a different subset of participants will complete FGDs (*n* = 6 FGDs, 2 per subpopulation type) at 12 months. Existing in-depth interview guides from prior research conducted among men and women in HIV-1 serodiscordant relationships and women at risk [33] have been adapted to focus on questions related to HIV-1 testing in the context of PrEP delivery. The in-depth interviews with participants in the intervention arms will provide a deeper understanding of participants’ HIVST experiences (including challenges, benefits, risks, and confidence in/preferences for blood versus oral fluid) and perceptions of less frequent PrEP clinic visits. The key informant interviews (*n* = 6–8) with PHRD clinic health care providers (e.g., counselors, nurses and clinicians) completed at month 12 will help in understanding the acceptance, barriers, facilitators, and confidence regarding HIVST in the context of PrEP. All in-depth interviews and FGDs will be conducted by experienced Kenyan social scientists, acting in the roles of facilitator and note taker, and audio recorded. All audio recordings will be transcribed and translated verbatim.

### Outcomes

The primary outcomes for this trial are PrEP adherence, PrEP continuation, and HIV-1 testing, measured at 6 months (Table 2).

**Table 2:** Primary trial outcomes

Outcome	Description
Pre-exposure prophylaxis (PrEP) adherence	The proportion of participants with any concentration of tenofovir diphosphate (TFV-DP) and emtricitabine triphosphate (FTC-TP) in a 3-mm punch from a dried blood spot (DBS) sample [12, 13]
Persistence in PrEP refills	The proportion of participants that return to the clinic to refill their PrEP medication
HIV-1 testing	The proportion of participants self-reported any HIV-1 testing (clinic- or home-based) in the past 6 months

#### PrEP adherence

PrEP adherence will be measured by testing for any concentrations of tenofovir diphosphate (TFV-DP) and emtricitabine triphosphate (FTC-TP) in a 3-mm punch from a DBS sample using liquid chromatography tandem mass spectrometry [12, 13, 47]. The PrEP levels in participants’ DBS samples will be standardized using participants’ hemoglobin measurements, which has been validated as an accurate means of interpreting PrEP levels [48].

#### Persistence in PrEP refills

Persistence in PrEP refills will be measured by calculating the proportion of participants that returned to the clinic to refill their PrEP medication using data from the PHRD clinic’s electronic pharmacy system. The ‘on-time’ window period for calculating the persistence in PrEP refills will be defined as 2 weeks prior to and 3 weeks following the scheduled follow-up date, but other window periods will also be used to calculate persistence in PrEP refills.

#### HIV-1 testing

Recent HIV-1 testing will be self-reported by participants at each study visit. In the follow-up surveys, participants will report how recently their last HIV-1 test was and by which means they were tested (e.g., clinic-based testing, HIVST, or other). HIVSTs (both used and unused) from participant in the intervention arms will be collected to validate if participants used the HIVST and correctly interpreted the results.

#### Secondary outcomes

A number of secondary outcomes will be measured at 6 and 12 months: 1) HIV-1 incidence; 2) social harms (self-reported verbal, physical, or emotional abuse by a sexual partner in the past 6 months); 3) the prevalence of depression (scores > 10 on the Patient Health Questionnaire-9 item depression scale, 0–27 points) [49]; 4) self-efficacy (using the General Self-Efficacy Scale [50], 10–40 points); 5) the number of sexual partners (in the past month); 6) inconsistent condom use (in the past month); 7) PrEP disclosure; and 8) HIV-1 testing preferences.

#### Adverse events

For the purposes of this study, only serious adverse events (SAEs) and adverse events related to PrEP (e.g., side effects or social harm) or self-testing (e.g., depression or social harm) will be documented and reported to the study’s DSMB. SAEs determined to be related to PrEP will result in temporary postponement of PrEP. In the case of temporary postponements, this will continue until the event is stabilized or resolved. If the event resolves, PrEP may be reinitiated at the discretion of the Investigator, with resumption of safety monitoring. Adverse events will be reported to relevant Institutional Review Boards (IRBs) according to clinical trial regulations, policies, and guidance [51]. Study staff will link participants reporting any adverse events with the appropriate services.

### Statistical analysis

#### Sample size considerations

The trial was powered using the PrEP adherence outcome (any detection of TFV-DP and FTC-TP in DBS samples) at 6 months. Based on published studies that measured PrEP adherence among HIV-1 serodiscordant couples [15] and women at risk of HIV-1 [43], PrEP adherence was estimated to be ~ 80% among PrEP users in both the standard-of-care and HIVST intervention arms at 6 months [15, 52]. A one-sided 95% confidence interval (common for noninferiority trials), 10% loss to follow-up, and 10% noninferiority margin were used to calculate power for this trial. With these parameters, it was determined that a sample size of 495 participants (*n* = 330 in the HIVST arms, *n* = 165 standard-of-care arm) would provide 80% power to detect noninferiority for 6-month PrEP adherence. A 10% noninferiority margin was selected for this trial because this is the reduction in PrEP adherence that might be tolerated to gain a programmatic efficiency in PrEP delivery through HIVST support. Two subgroup analyses, with sample sizes of 330 participants, are planned for this trial: 1) men and women in HIV-1 serodiscordant relationships (which will include 165 men and 165 women); and 2) women at risk of HIV-1 (which will include the 165 in HIV-1 serodiscordant relationships plus the 165 at risk but not necessarily in a serodiscordant relationship). These primary subgroup analyses have 80% power to rule out a 12% decrease in PrEP adherence (i.e., a slightly greater noninferiority margin).

#### Quantitative data

All analyses comparing randomization arms will be performed using the intention-to-treat method. The primary comparison will be self-testing versus clinic-based testing; the two self-testing modalities will be analyzed together because we hypothesize that the effect on PrEP adherence and other outcomes relates to the use of self-tests and frequency of follow-up, not the self-test modality per se. To measure effect size estimates, modified Poisson regressions will be used for binary outcomes and multivariable linear regression models will be used for continuous outcomes. Models comparing randomized arms will include the study arm as the primary predictor in the model, and will adjust only for study population (males in an HIV-1 serodiscordant relationship, females in an HIV-1 serodiscordant relationship, and females not in an HIV-1 serodiscordant relationship). Supplemental, adjusted analyses also will be performed where potential confounders are found to differ at baseline. Potential confounders will be based on our prior work assessing correlates of PrEP use: demographics (e.g., gender, age, educational level), sexual behaviors (e.g., condom use, outside relationships), medical status (e.g., depression), and beliefs (e.g., risk perception, PrEP efficacy). Analyses will be done to test effect modification by HIV-1 serodiscordant relationship status and gender. Significance will be assessed using a two-tailed test at the 0.05 level.

#### Qualitative data analysis

Two Kenyan social scientists will review the transcripts from the in-depth individual interviews, FGDs, and key informant interviews for completeness and initial theme generation. Coding and analysis will be performed in Dedoose (Los Angeles, California, USA), using an inductive approach informed by grounded theory [53]. The results of the coding will be reviewed for consistency of text segmentation and code application, using continued inter-coder agreement. The coders will review inconsistent coding results until consensus is reached. Codes will then be grouped together into themes through consensus among coders [54, 55].

### Confidentiality

To protect participants’ confidentiality, no identifying information will be collected during the quantitative surveys or qualitative interviews and all collected data will be de-identified. A unique study identification number will be used to link data from an individual participant over time.

### Dissemination

As soon as the trial results are available and finalized, they will be shared with participants and local Kenyan stakeholders through meetings and a one-page flyer that summarizes the results in clear language. The trial results will be analyzed and written up for presentation at a scientific conference and publication in a peer-reviewed scientific journal. The trial is registered with Clinicaltrials.gov (NCT03593629) [34] and the results will be updated there in a timely fashion. A special meeting with members from the Kenyan MOH (including members from the PrEP and HIV-1 testing Technical Working Groups) will be hosted to share the trial results so that the findings can support policy for PrEP delivery in Kenya.

## Discussion

This innovative trial tests if HIVST can streamline PrEP delivery for men and women in HIV-1 serodiscordant relationships and women at risk of HIV-1 through decreasing the frequency of PrEP clinic visits by having HIVST at home to replace HIV-1 testing at clinics. The trial uses both oral-fluid and blood-based HIVST to determine if the two HIVST modalities might have different use, acceptability, costs or preferences. Maximizing access and minimizing costs of delivery are key challenges for optimizing the public health impact of PrEP for HIV-1 prevention [16–18], particularly in resource-constrained settings. In Africa, PrEP will be added to an already overburdened health infrastructure, and thus the ability of public health systems to afford PrEP will necessitate making its delivery cost-effective and time-efficient. PrEP delivery programs will need to be cost-sensitive to staffing needs (e.g., frequent clinic visits); moreover, patients may not continue PrEP if their costs (e.g., travel to/waiting in clinics) are high. HIV-1 testing is central to PrEP delivery and the opportunity of HIVST—a recent innovation [56]—to improve HIV-1 prevention has not yet been fully realized.

There are a number of potential benefits and concerns with this trial of HIVST to support PrEP delivery. One major benefit is that reducing the number of PrEP clinic visits through HIVST might save costs for both healthcare clinics delivering PrEP and individuals using PrEP. Another benefit is that the reduced number of PrEP clinic visits may even improve PrEP adherence if PrEP users are likely to miss interim visits for reasons including cost and time associated with traveling to PrEP clinics or stigma associated with PrEP clinic visits [57]. One concern with this trial, however, is that the long duration between PrEP visits (6 months) and reduced contact with PrEP providers might instead reduce adherence among PrEP users because contact with the health system is needed to stay motivated and/or be supported to continue with good adherence. In addition, there are concerns that participants in the HIVST intervention arms might be at greater risk of experiencing intimate partner violence if, for example, sexual partners find out about HIV-1 status through HIVST [58]. All participants will be carefully screened for incidents of intimate partner violence at each study visit and will be linked to existing services if they seem at risk, as this study team has done in other PrEP and HIVST studies.

In conclusion, the findings from this trial can help to understand how HIVST, a new technology, can improve the health of individuals and support health systems in sub-Saharan Africa. A number of efficacious HIV-1 prevention interventions exist, but access and adherence to these interventions in low-resource settings can be a challenge. Developing innovative ways to improve delivery models for PrEP and other HIV-1 prevention interventions is imperative. The results of this trial are expected to inform policy aimed at improving the efficiency of PrEP implementation and scale-up in Kenya and other sub-Saharan African countries.

## Trial status

The first trial participant was enrolled and randomized on 28 May 2018; the last trial participant is expected to be enrolled and randomized around December 2019. The trial was first submitted to ClinicalTrials.gov on 3 April 2018, but a delay in the review pushed the registration to 20 July 2018. All relevant Ethics Review Committees have accepted amendments to the study protocol, including the collection of hemoglobin to confirm measurements of PrEP adherence measured in DBS samples on 9 September 2018.

## Additional file

Additional file 1: SPIRIT 2013 checklist: recommended items to address in a clinical trial protocol and related documents. (DOC 123 kb)

## Data Availability

The study investigators will provide access to the trial protocol, trial data and statistical code upon request.

## Abbreviations

3TC: Lamivudine
ART: Antiretroviral therapy
DBS: Dried blood spot
DSMB: Data Safety Monitoring Board
FGD: Focus group discussion
FTC: Emtricitabine
HIV-1: Human immunodeficiency virus-1
HIVST: HIV-1 self-testing
IRB: Institutional Review Board
MOH: Ministry of Health
PHRD: Partners in Health and Research Development
PrEP: Pre-exposure prophylaxis
SAE: Serious adverse event
SPIRIT: The Standard Protocol Items: Recommendations for Interventional Trials
TDF: Tenofovir disoproxil fumarate
TFV-DP: Tenofovir diphosphate
